# The Health and Well-Being of Transgender Australians: A National Community Survey

**DOI:** 10.1089/lgbt.2020.0178

**Published:** 2021-01-12

**Authors:** Ingrid Bretherton, Emily Thrower, Sav Zwickl, Alex Wong, Daria Chetcuti, Mathis Grossmann, Jeffrey D. Zajac, Ada S. Cheung

**Affiliations:** ^1^ Department of Medicine (Austin Health), The University of Melbourne, Victoria, Australia.; ^2^ Department of Endocrinology, Austin Health, Heidelberg, Victoria, Australia.

**Keywords:** barriers to care, gender-affirming endocrine care, gender-affirming surgical care, gender dysphoria, transgender

## Abstract

***Purpose:*** Transgender, including gender diverse and nonbinary (trans), people experience significant health disparities. We aimed to better understand the health status and needs of Australian trans people to guide resources and health and well-being programs.

***Methods:*** This anonymous, cross-sectional online survey utilized nonprobability snowball sampling of Australian adults (18 years and over) who self-identified as trans between September 2017 and January 2018. This descriptive study assessed demographic data, community views on access to health care, health burden, access to health resources, and priorities for government funding in transgender health.

***Results:*** Of 928 participants, 37% reported female, 36% reported male, and 27% reported nonbinary gender identities. Despite 47% having tertiary qualifications, the unemployment rate was 19%, with 33% reporting discrimination in employment due to being trans. Discrimination in accessing health care was reported by 26% and verbal abuse and physical assault were reported by 63% and 22%, respectively. Lifetime diagnosis of depression was reported by 73% and anxiety by 67%. Sixty-three percent reported previous self-harm and 43% had attempted suicide. Autism spectrum disorder and attention-deficit/hyperactivity disorder were reported by 15% and 11%, respectively. The most preferred method of receiving health information was through online resources, with the most popular source being Reddit, an online peer discussion board. Better training for doctors in trans health issues was the top priority for government funding.

***Conclusions:*** Barriers, including widespread discrimination and unemployment, contribute to health inequity and prevalent mental health conditions. Better training for health professionals in the provision of safe, gender-affirming and general health care for trans people is urgently required.

## Introduction

The number of transgender, including gender diverse and nonbinary (trans), individuals seeking gender-affirming health care worldwide is rising,^1^ yet global studies have demonstrated that trans people face many barriers to accessing health care, including discrimination^2^ and the inability to find doctors willing to provide care,^3^ as well as high rates of depression and attempted suicide.^1,4^ Mental health distress is driven, in part, by barriers to accessing health care as well as by discrimination.^1,5–8^ In addition, co-occurring autism spectrum disorders (ASD) and attention-deficit/hyperactivity disorder (ADHD) may also be more prevalent among trans individuals for unclear reasons, with difficulties with attention or social interaction potentially posing greater barriers by affecting the ability to understand health information or engage in clinical care.^1,9^

There are little data describing the health of the Australian adult trans population. Due to a lack of population data, it is unknown how many Australians identify as trans. A nonpeer-reviewed publication described very high levels of mental health conditions, particularly depression and anxiety syndromes, poor quality of life, and high rates of discrimination among Australian trans adults in 2013 (Ref.^10^). Similarly, high rates of mental health conditions were observed in trans adults attending specialized gender affirmation clinics in the state of Victoria; however, these findings may not be generalizable.^1^

Australia's universal health care system provides free or low-cost, government-subsidized general health services, including general or specialist consultations, pathology collection, and medications, including gender-affirming hormones. However, in regions with fewer specialized gender services, access to low-cost options may be limited.

Access to gender-affirming interventions in Australia typically follows one of two pathways; either a formal assessment and approval by a mental health professional as per the World Professional Association for Transgender Health Standards of Care^11^ or an alternative informed consent model of care where a decision to commence gender-affirming hormones is shared between a primary care general practitioner and a trans individual without mandating a formal mental health review.^12^ Due to a lack of publicly funded gender-affirming surgery, this is provided almost entirely in the private health sector, which carries significant out-of-pocket costs.

This community-based survey sought to better understand the health needs of Australian trans individuals to direct local health resources to best meet health care needs. We hypothesized that transgender individuals have significant barriers to accessing health care, including socioeconomic disadvantage, high burden of co-occurring mental health conditions, and discrimination. The aim of this descriptive study was to assess the sociodemographic characteristics and medical and mental health conditions affecting adult trans Australians; to obtain views on health burden, ability to access health care, and ability to access health resources; and to understand community views on funding priorities for trans health.

## Methods

This anonymous community survey utilized a nonprobability snowball sampling approach to survey trans Australian adults aged 18 years and over using an online survey platform (SurveyMonkey, Inc., USA) between September 1, 2017, and January 31, 2018. The full survey is listed in Supplementary Appendix SA1. Participants were recruited through the Trans Health Research group Facebook page and the study also was promoted at the Australian and New Zealand Professional Association for Transgender Health Biennial Meeting in Sydney, Australia, in September 2017 and at the Midsumma LGBTIQ+ Festival in Melbourne, Australia, in January 2018. Written informed consent was not possible given the anonymous online design; however, the survey preamble outlined that completion of the survey implied consent. The survey link was available as a URL and did not require access to a specific social media account. The study was approved by the Austin Health Human Research and Ethics Committee (HREC/17/Austin/372).

Inclusion criteria were assessed through a positive response to three screening questions: (1) residency in Australia; (2) identification as trans or had previously identified as such; and (3) aged 18 years or over. The inclusion of those who had previously identified as trans was intended to include those who identified as their affirmed gender (male or female) rather than with the term transgender. Individuals were eligible to complete the survey on one occasion only and duplicate responses from the same internet protocol address were excluded. All included individuals had discordance between their assigned sex at birth and their gender identity. Other than the initial screening questions, all subsequent survey questions were optional.

### Demographic data

Participants' birth years and postcodes were obtained. Postcodes were coded as per the Australian Standard Geographical Classification–Remoteness Area (RA) coding^13^ to one of five groups; RA1 (inner cities) to RA5 (very remote). Participants were asked to select their sex assigned at birth (male, female, or intersex) and their gender identity (see Table 1 for options). To enable meaningful statistical analyses, gender identities were then further categorized into three groups: trans man/trans male/trans masculine and male gender identities were coded as male identities; trans woman/trans female/trans feminine and female were coded as female identities; and gender nonbinary, gender queer, gender neutral, gender fluid, intersex, and agender were coded as nonbinary gender identities. Those who selected “other” also entered free text and were reclassified accordingly. Formal education, requirement for government financial assistance, and employment status were assessed (responses as outlined in Table 1). Participants were able to select more than one employment status. To reflect engagement with the workforce, if two options were selected, individuals were classified in the group that reflected the most workforce engagement. For example, if a person was a student and casually employed, they were classified as casually employed.

**Table 1.** Sociodemographic Parameters of the Participants

Parameter	Number of responses received	Frequency,* n *(%)
State of residence	911	
Victoria		282 (31)
New South Wales		195 (21)
Queensland		143 (16)
Western Australia		126 (14)
South Australia		92 (10)
Tasmania		37 (4)
Australian Capital Territory		34 (4)
Northern Territory		2 (<1)
Age group (years)	928	
18–24		289 (31)
25–29		216 (23)
30–39		193 (21)
40–49		125 (13)
50–59		71 (8)
60–69		30 (3)
70–79		4 (<1)
Sex assigned at birth	928	
Female		520 (56)
Male		403 (43)
Intersex		5 (1)
Gender identity	928	
Male		91 (10)
Female		140 (15)
Trans man/trans male/trans masculine		239 (26)
Trans woman/trans female/trans feminine		202 (22)
Gender nonbinary		133 (14)
Gender queer		41 (4)
Gender neutral		11 (1)
Gender fluid		19 (2)
Intersex		2 (<1)
Agender		20 (2)
Other		30 (3)
Education level	928	
Never attended school		1 (<1)
Primary school		0
Some high school		98 (11)
Completed high school		222 (24)
Trade/technical certificate or apprenticeship		170 (18)
University or tertiary qualifications		437 (47)
Employment status	928	
Employed on a full-time basis		274 (30)
Employed on a part-time or casual basis		224 (24)
Home duties full-time		13 (1)
Student		176 (19)
Retired		20 (2)
Unemployed		177 (19)
Other (free text)		44 (5)

### Access to health care and health burden

Current smoking and past 12-month illicit drug use were self-reported, and self-perception of overall health was evaluated (responses available outlined in Table 2). Participants were asked about their access to various types of health care providers, including availability of general practitioners and their confidence in discussing health issues of concern with their treating doctor. As discrimination has been identified as a barrier to health care in previous surveys,^14^ participants were asked if they had perceived discrimination in employment, housing, accessing health care, and government services and/or whether they had experienced physical assault, verbal abuse, and domestic violence because of their gender identity. trans individuals were asked whether they had experienced any difficulty accessing hormonal treatment (such as the inability to find a doctor who is willing to prescribe, financial costs of prescriptions, financial costs of doctor's appointments, or other [specify]). Participants were also asked if they had taken any hormonal treatments without a prescription.

**Table 2.** Access to Health Care and Health Burden

Parameter	Number of responses received	Frequency,* n *(%)
Self-perception of overall health	907	
Excellent		86 (9)
Very good		224 (25)
Good		401 (44)
Poor		171 (19)
Very poor		25 (3)
Health care providers utilized^a^	928	
GP		779 (84)
Psychologist		631 (68)
Psychiatrist		508 (55)
Endocrinologist		413 (45)
Surgeon		298 (32)
Nurse		235 (25)
Speech pathologist		117 (13)
Gender clinic within a hospital		103 (11)
Gynecologist		87 (9)
None		89 (10)
Other (free text)		32 (3)
Discrimination^a^	927	
Discrimination in employment		304 (33)
Discrimination in accessing health care		244 (26)
Discrimination in government services		149 (16)
Discrimination in housing		95 (10)
Verbal abuse		584 (63)
Physical assault		200 (22)
Domestic violence		133 (14)
Difficulty accessing hormonal treatment^a^	905	
None		372 (41)
Unable to find a doctor to prescribe		148 (16)
Financial costs of prescriptions		124 (14)
Financial costs of doctor's appointments		156 (17)
Pathway to accessing hormones was too difficult		284 (31)
Other (specify)		100 (11)
Views on informed consent—Should trans people undertake a formal mental health practitioner assessment?	913	
Yes, in all cases		285 (31)
Yes, but only in some circumstances		392 (43)
No		187 (20)
Unsure		48 (5)
Masculinizing hormone treatments in birth-assigned females^a^	509	
None		191 (38)
Testosterone injections		267 (53)
Testosterone creams, gels, or patches		45 (9)
Testosterone implants		2 (<1)
GnRH analogs		2 (<1)
Progestins		4 (<1)
Other		7 (1)
Feminizing hormone treatments in birth-assigned males^a^	402	
None		75 (19)
Estradiol oral tablets		205 (51)
Estradiol transdermal patches		56 (14)
Estradiol gels		33 (8)
Estradiol implants		52 (13)
Combined oral contraceptive pill		14 (3)
Spironolactone		130 (32)
Cyproterone acetate		106 (26)
Bicalutamide		1 (<1)
GnRH analogs		2 (<1)
Progestins or micronized progesterone		63 (16)
Other (i.e., finasteride or estradiol injections)		11 (3)
Overseas surgery	914	
Yes		72 (8)
No		841 (92)
Unsure/prefer not to say		1 (<1)
Medical conditions	914	
Depression		663 (73)
Anxiety		613 (67)
Fractures (broken bone)		191 (21)
Autism spectrum or Asperger's syndrome		137 (15)
ADHD		96 (11)
Bipolar disorder		75 (8)
Diabetes mellitus		25 (3)
Cancer		19 (2)
Blood clots (pulmonary embolus or deep vein thrombosis)		16 (2)
Liver disease		13 (1)
Stroke		11 (1)
HIV/AIDS		5 (<1)
Ischemic heart disease		4 (<1)
Emphysema		3 (<1)
Kidney or renal disease		3 (<1)
None of the above options selected^b^		136 (15)

^a^ Multiple responses were allowed for this question, so total responses do not sum to 100%.

^b^ None was not an option in the survey but was presumed if no medical conditions were selected but answers were completed to the remaining questions in Section 2: Your Health of the survey.

ADHD, attention-deficit/hyperactivity disorder; GnRH, gonadotropin-releasing hormone; GP, general practitioner; trans, transgender, including gender diverse and nonbinary.

To assess the community's value of mental health assessments before commencing gender-affirming hormonal treatment, participants were asked “Do you feel that a mental health assessment for trans and gender diverse individuals should be performed prior to accessing hormonal treatment?” Assessment of access to and desire for gender-affirming hormonal and surgical treatments and previous medical and mental health conditions relied on self-reporting, and no specific diagnostic tools were used. History of self-harm or attempted suicide was also ascertained.

### Access to health resources and priorities for government funding

Preferred methods (i.e., social media, online resources, videos, forums, and print) of receiving health information were assessed, including involvement in support groups and websites used to locate information on trans health. Desire for local, Australian-based, trans health resources was also determined. Participants selected the areas of priority to which they thought resources should be directed (education about gender diversity, gender clinics, support groups, trans advocacy groups, counseling, better training for doctors in trans issues, transgender medical research, psychology/psychiatry services, or other [free text]). Qualitative analysis results of several open-ended questions regarding health issues of concern have been reported separately.^15^

### Statistical analysis

Statistical analysis was performed using SPSS Statistics, version 23 (IBM Corporation, Armonk, NY). Descriptive frequencies are reported and medians (interquartile range) are reported for non-normally distributed data.

## Results

The survey social media post was shared by 275 individuals and transgender support groups on the social media site Facebook. A total of 964 responses to the survey were obtained. After excluding duplicates from the same IP address, blank surveys, or those that did not meet the inclusion criteria (based on the previously described screening questions), 928 eligible responses remained.

### Sociodemographic data

As shown in Table 1, responses were received from every Australian state and territory. The greatest number of participants (*n* = 282, 31%) resided in Victoria. Eighty-three percent (*n* = 752) of those that responded resided in inner city areas (RA1). Median age was 28 years (interquartile range 23–39). Thirty-seven percent (*n* = 342) reported female identities, 36% (*n* = 330) reported male identities, and 27% (*n* = 256) reported nonbinary gender identities. Participants had high levels of education, with 47% (*n* = 437) holding a university qualification. The unemployment rate was 19% (*n* = 177). The majority (*n* = 376, 57%) reported receiving some form of government financial assistance.

### Access to health care and health burden

Table 2 outlines responses describing access to health care and health burden. Current smoking in 15% (*n* = 141) of participants is comparable with national data indicating that 11.6% of Australian adults reported smoking cigarettes daily.^16^ Illicit drug use was high, with 33% (*n* = 305) of respondents reporting use of illicit drugs in the past 12 months and is approximately double the general Australian population rate of illicit drug use of 16.4% in the preceding 12 months in 2019 (reported in people aged 14 years and over).^17^ Nearly 80% (*n* = 711) described at least good health and 80% (*n* = 732) had a regular family doctor or general practitioner. When asked if individuals had ever experienced any difficulty accessing hormonal treatment, 41% (*n* = 372) selected “none.” A third (*n* = 284) reported that the pathway to accessing hormones was too difficult. Discrimination because of gender identity was widespread, with 33% (*n* = 304) reporting discrimination related to employment and 26% (*n* = 244) related to accessing health care. Verbal abuse because of their trans status was reported by 63% of respondents and physical assault because of their trans status was reported by 22%.

**FIG. 1.**
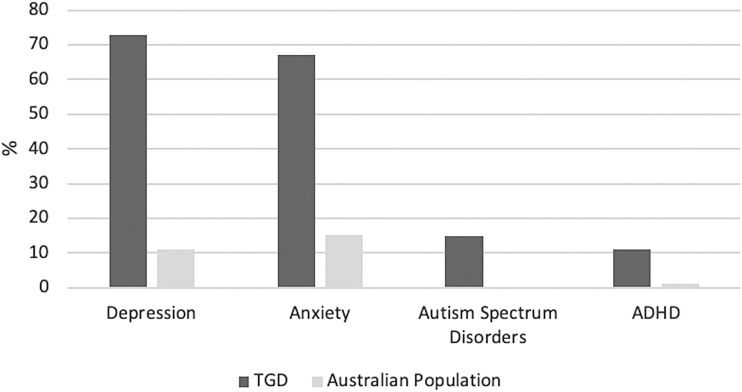
Self-reported diagnoses in trans individuals versus the Australian population (age-matched).^18–20^ ADHD, attention-deficit/hyperactivity disorder; trans, transgender, including gender diverse and nonbinary.

There were mixed responses to the need for a formal mental health assessment prior to commencement of hormonal therapy and it is acknowledged that wording of this question may have contributed to ambiguity (Table 2). There was a very high prevalence of self-reported depression and anxiety as well as ASD and ADHD (Fig. 1).^18–20^ Intentional self-harm was reported by 63% (*n* = 577) of participants and 43% (*n* = 394) reported having previously attempted suicide.

Gender-affirming surgical interventions are summarized in Table 3. Genital reconstruction surgery was the most common procedure undertaken by those assigned male at birth (*n* = 71, 18%); however, a further 64% (*n* = 243) desired this surgery in the future. The most frequent procedure undertaken by those assigned female at birth was bilateral mastectomy or chest reconstruction (*n* = 159, 31%). Similarly, a further 58% (*n* = 297) desired this procedure in the future.

**Table 3.** Access to and Desire for Gender-Affirming Surgery

	Total number of responses	Have had,* n *(%)	Want someday,* n *(%)	Don't want,* n *(%)
Surgical procedures in birth-assigned males (*n* = 403)				
Breast augmentation	362	32 (9)	196 (54)	134 (37)
Genital reconstruction surgery	384	71 (18)	243 (64)	70 (18)
Facial feminization surgery	372	23 (6)	235 (63)	114 (31)
Voice surgery	348	6 (2)	149 (43)	193 (55)
Surgical procedures in birth-assigned females (*n* = 520)				
Chest surgery/mastectomy	511	159 (31)	297 (58)	55 (11)
Genital reconstruction surgery	481	10 (2)	213 (44)	258 (54)
Voice surgery	405	1 (<1)	15 (4)	389 (96)

Percentages are rounded to whole numbers.

### Access to health resources and priorities for government funding

The most preferred method of receiving health information was through online resources (*n* = 400, 50%) (Table 4). Forty-three percent (*n* = 369) of participants used existing online sources for health information. The most popular source reported in this study was Reddit, an online discussion board with user-generated content, followed by Facebook, Susan's Place, FtM Australia, Wikipedia, YouTube, and Tumblr. The majority (95%, *n* = 814) supported the development of a comprehensive online website with local, Australian-based, trans health resources, and 89% of the participants (*n* = 768) used social media daily. Better training for doctors in trans issues was the most frequently selected priority for government funding (32%, *n* = 267); complete responses are listed in Table 4.

**Table 4.** Access to Health Resources and Priorities for Government Funding

Parameter	Number of responses received	Frequency,* n *(%)
Most preferred method of receiving health information	799	
Online (websites and e-mail, etc.)		400 (50)
Social media (e.g., Facebook)		150 (19)
Videos or podcasts		57 (7)
Telephone contact		43 (5)
Hardcopy print materials (e.g., brochures)		41 (5)
Small local community talks/seminars		39 (5)
Apps (on mobile devices)		35 (4)
Online group forums (e.g., webinars)		24 (3)
Larger group gatherings (e.g., conferences)		10 (1)
Social media use	859	
Daily		768 (89)
Couple of times a week		55 (6)
Occasionally (e.g., once a week)		19 (2)
Rarely (e.g., once a fortnight)		10 (1)
Not at all		7 (<1)
Top priority for government funding	824	
Better training for doctors in trans issues		267 (32)
Gender clinics		205 (25)
Education about gender diversity (i.e., community or schools)		197 (24)
Trans or gender-related medical research		83 (10)
Psychology or psychiatry services		32 (4)
Support groups		18 (2)
Trans advocacy groups		14 (2)
Counseling		8 (1)
Other (free text)		0

## Discussion

This large community-based survey involving 928 participants described persistent, concerning health statistics among trans Australian adults: high rates of self-reported mental health morbidities, such as anxiety and depression, as well as high rates of self-reported self-harm (63%) and attempted suicide (43%). There were widespread experiences of discrimination, especially in health care settings (26%). Moreover, a majority of the participants had experienced verbal abuse (63%), with fewer reporting physical assault (22%) because of their trans status. There were barriers to employment (19% unemployed) despite high levels of tertiary education. Additional barriers to accessing health care existed, such as difficult ambiguous pathways for accessing gender-affirming hormonal therapy; difficulty finding doctors to prescribe treatment; and the potentially high, out-of-pocket financial costs of surgical care. Although the use of gender-affirming hormone therapy was common, significant difficulties existed in accessing gender-affirming surgery. Even though most of the participants accessed health information from peer-generated online websites, there was support for development of reliable, local health resources. Better training for doctors in trans health issues was highlighted as the top priority for government funding by 32% of participants.

### Sociodemographic data

We observed a breadth of gender identities in the trans community across Australia with approximately equal thirds of the participants having female, male, and nonbinary identities. This contrasts with historical reports that the prevalence of trans female individuals outnumbered trans male individuals.^21^ The high proportion of people with nonbinary gender identities is consistent with rates observed in our primary care clinics in Australia^1^ and may reflect increasing societal views that challenge binary gender stereotypes.

The unemployment rate of 19% was three times that of the Australian general population rate of 5.5% in May 2018 and well above the youth unemployment rate (12.2%).^22^ Notably, 33% of respondents perceived discrimination in employment. Unemployment may also occur due to difficulty with name and identity documents, discrimination in basic housing and health care,^5^ and the impact of mental health conditions such as depression and anxiety on an individual's ability to seek or maintain employment. Conversely, levels of depression and anxiety may be higher due to unemployment.^23^

### Access to health care and health burden

Similar to prior reports,^5^ discrimination in all aspects of life was frequently reported by trans Australians, which is not only harmful but also perpetuates inequity. Most concerning is that safe access to health care, which should be accessible to all, is not a reality for trans Australians and this is supported by the participants' selected top priority for government funding being better training for doctors in trans health issues. Access to surgery is a major challenge in Australia, with (anecdotally) few surgeons experienced in providing gender-affirming surgery. Moreover, surgery is predominantly provided in the private health system, which is associated with prohibitive financial costs. There is a need for education and training to target the number of surgeons providing gender-affirming surgery.

Self-reported depression and anxiety were highly prevalent in ∼70% of individuals, as were self-reported diagnoses of ASD and ADHD (Fig. 1). These are consistent with data from individuals attending specialized gender clinics^1^ as well as from international reports.^24^ Notably, a diagnosis of ADHD in childhood is associated with a higher risk of having at least one mental health condition and a higher risk of death by suicide.^25^ As positive screening tools for ASD may reflect elevated social anxiety experienced by trans people, data describing the coexistence of ASD are conflicting and further research is needed.^9^

The most concerning data are the self-reported self-harm and attempted suicide rates, a reflection of the severe distress and despair that many trans individuals have faced. These suicidality rates are much higher than the lifetime prevalence of suicide attempts in Australian adults (3.3%).^26^ Our Australian data mirror findings in the U.S. National Transgender Discrimination Survey of 6450 trans Americans, which first highlighted widespread discrimination in many aspects of life, including double the rate of unemployment; 19% being refused medical care due to their trans status; and 41% of suicide attempts (compared with 1.6% of the general population).^27^ Lack of acceptance in the community and, at times, among health professionals leaves few resources for trans individuals to access help and support. This is a significant public health concern and there is an urgent need for a coordinated and combined suicide prevention response.

### Health resources and priorities for government funding

The top priority for government funding was better training in trans health issues for doctors. Although greater awareness of and more coordinated training in trans health need to occur, in response to findings from this study, an evidence-based local position statement was published regarding the hormonal management of trans adults to provide a point-of-care resource for doctors caring for trans individuals.^12^ In response to the community desire for Australian-based trans health information, we contributed to the development of trans community-led online health resources (Trans Health Research and TransHub).^28,29^

### Limitations

There are multiple limitations to this study. The online-based recruitment may explain why a greater proportion of responders were younger individuals and may not accurately reflect the views of the older trans community. There may be self-selection bias and not all areas of Australia were equally represented as recruitment was not targeted. There was a predominance of respondents from southeastern states, which may be related to physical promotion of the study at one event in Victoria and one in New South Wales. However, distribution of respondents was similar to a previous 2013 Western Australian-based survey.^10^ Ethnicity data were not collected, so we were unable to ascertain if this was a factor associated with additional barriers when accessing health care. Medical conditions were self-reported, and we were not able to utilize any diagnostic measures to confirm diagnoses. Furthermore, we did not gauge temporal trends in diagnoses and did not distinguish current from past medical conditions, which may be particularly relevant in the interpretation of the prevalence of ADHD. Participants were asked whether a mental health assessment for trans and gender diverse individuals should be performed prior to accessing hormonal treatment. There was likely a response bias in favor of the mental health assessment model as we did not make it clear that we were referring to a formal mental health assessment by a psychologist or psychiatrist rather than by the primary care physician in the wording of this question.

However, this survey provided a platform for participants to express their views anonymously, which potentially facilitated the expression of more honest responses than a face-to-face interview or government statistics form. The fact that many of our findings, although self-reported (such as rates of self-harm), replicate those from prior similar studies conducted with other transgender populations supports both the validity and the generalizability of our findings. Despite the limitations, this is one of the largest published studies of adult trans individuals in the Australian population and provides valuable insight on the status of health and health needs of a traditionally marginalized community that is underrepresented in research.

## Conclusions

This large community survey highlights a myriad of challenges faced by trans adult Australians, including discrimination, abuse, unemployment, and inability to find doctors to access general health care and gender-affirming care. Reducing the high attempted suicide rate and burden of mental health conditions needs to be prioritized. The participants in this study identified the training of doctors in trans health as a priority. This should be one of the first steps to ensure that basic health needs are met. Urgent action is required from a policy perspective to address the concerning health disparities described herein and to ensure that all trans people are safe and empowered to live a life without barriers.

## Supplementary Material

Supplemental data

## Supplementary Material

Supplementary Appendix SA1
