# Supplementary material for: The Health and Well-Being of Transgender Australians: A National Community Survey
Source: LGBT Health. 2021 Jan 12;8(1):42–9. doi: 10.1089/lgbt.2020.0178 (PMC7826417; doi:10.1089/lgbt.2020.0178)
Supplement: Supplemental data [file Supp_Appendix1.docx]

**SUPPLEMENTARY APPENDIX SA1**

**Community Survey**

**We invite you to have your say on trans health in Australia.**

**The purpose of this study**

This survey is to help understand more about transgender healthcare in Australia. This is part of a series of surveys aimed at transgender and gender diverse individuals, as well as healthcare providers in Australia. A previous report in 2013 found that the state of trans health in Australia was in a state of crisis. Similar reports have been found in children and youth recently. High rates of mental health distress was found, in part driven by barriers to accessing healthcare and discrimination. The aim of this 2017-2018 survey is to better understand the health needs of trans and gender diverse adult individuals in Australia to direct health resources, research directions and inform how we can meet the care needs of adult trans and gender diverse individuals.

The survey should take around 10 minutes to complete and you will not be identified in any way by completing it as your responses will remain anonymous. Your participation in this survey is entirely voluntary and you may discontinue at any time. Completing the survey implies consent for your information to be used for the purpose as outlined above. By clicking ‘next’ you confirm you are 18 years or over and have read the information above.

This survey is intended for individuals who identify as trans or gender diverse or who have previously identified as trans or gender diverse. This survey is designed to help us understand more about transgender individuals, as well as the barriers they face when accessing healthcare services.

**Taking part in this study**

You must be aged over 18 to take part in this survey. You do not need to complete a formal consent form to take part in this study. By completing this survey, you are consenting that your answers will be used in this study. All responses are anonymous. This study has been approved by the Austin Health Human Research Ethics Committee (Reference number HREC/17/Austin/372)

**Risks of this project**

If you feel uncomfortable answering any questions you may choose not answer a particular question or choose not to complete the survey. This survey asks sensitive questions about mental health. If this survey raises concerns for you or someone you know please see your doctor or contact the following organisations for support:

Lifeline on 13 11 14 or https://www.lifeline.org.au
Beyond Blue 1300 22 4636 or https://www.beyondblue.org.au QLife 1800 184 527 https://qlife.org.au/

**Further information and who to contact**

The person you may need to contact will depend on the nature of your query:

Clinical contact person
Dr Ingrid Bretherton
PhD Candidate
Telephone: 9496 2486
Email: ibretherton@student.unimelb.edu.au

If you have any complaints about any aspect of the project, the way it is being conducted or any questions about being a research participant in general, then you may contact:

Complaints Officer
Telephone: (03) 9496 4090 or (03) 9496 3248

Email: ethics@austin.org.au

Thank you for participating in this study. Please feel free to share the link to this survey with any of your trans or gender diverse networks.

**Section 1: About you**

The following survey is intended for people that identify as transgender or gender diverse or individuals who have previously undergone gender transition. Please select the answers that most closely reflect your views. Thank you.

1. Do you currently identify or have you previously identified as transgender or gender diverse?

🞏 Yes

🞏 No

1. Do you live, work or receive healthcare in Australia?

🞏 Yes

🞏 No

1. In what year were you born? (enter 4-digit birth year; for example, 1976) ________
2. What is the postcode of the suburb that you currently live in? ________
3. How would **you** describe your current gender identity?

🞏 Male

🞏 Female

🞏 Trans Male / Trans Masculine / Trans Man

🞏 Trans Female / Trans Feminine / Trans Woman

🞏 Gender non-binary

🞏 Gender queer

🞏 Gender neutral

🞏 Gender fluid

🞏 Intersex

🞏 Agender

🞏 Other: If none of the above adequately describe your gender identity please enter your answer as free text: ___________________

1. What was your birth-assigned sex or biological sex assigned to you at birth?

🞏 Female

🞏 Male

🞏 Intersex

1. What is your country of birth?
   🞏 Australia

🞏 New Zealand
🞏 UK/ Ireland
🞏 Vietnam

🞏 Italy

🞏 China

🞏 Greece

🞏 Netherlands

🞏 Japan

🞏 Mexico

🞏 India

🞏 Spain

🞏 United States

🞏 Germany

🞏 Malaysia

🞏 Other (please specify) ______________________________

1. What is your highest level of education?

🞏 Never attended school
🞏 Primary school
🞏 Some high school

🞏 Completed high school

🞏 Trade/ technical certificate or apprenticeship

🞏 University qualification

1. How would you describe your current employment?

🞏 Employed on full-time basis
🞏 Employed on part time or causal basis
🞏 Unemployed

🞏 Home duties full time

🞏 Student

🞏 Retired

🞏 Other (please specify)

1. Do you currently receive any government assistance (such as Centrelink)?

🞏 Yes

🞏 No
🞏 Prefer not to say

**Section 2: Your Health**

1. How would you describe your overall health?

🞏 Excellent

🞏 Very good

🞏 Good

🞏 Poor

🞏 Very poor

1. Do you currently smoke cigarettes (within the past week)?

🞏 Yes

🞏 No

🞏 Unsure/ Prefer not to say

1. Have you taken any illicit recreational drugs within the past 12 months? (This includes marijuana, heroin, ice, cocaine etc)

🞏 Yes

🞏 No

🞏 Unsure/ Prefer not to say

1. Do you have a regular family doctor/ general practitioner (GP)?

🞏 Yes

🞏 No

🞏 Unsure/ Prefer not to say

1. Which of the following healthcare providers have you seen at any point for gender-related care? (Select all that apply)

🞏 GP

🞏 Nurse

🞏 Psychologist

🞏 Psychiatrist

🞏 Endocrinologist

🞏 Gynaecologist

🞏 Surgeon

🞏 Speech pathologist

🞏 Gender clinic within a hospital

🞏 None, I haven't seen any health professional for gender- related care

🞏 Other (please specify) ________________________________

1. Which gender-affirming hormonal treatments (if any) do you currently take? (Select all that apply)

🞏 None

🞏 Testosterone injections (Reandron,Primoteston, Sustanon)

🞏 Testosterone implants
🞏 Testosterone creams, gels or patches
🞏 Oestrogen tablets (i.e. Progynova)

🞏 Oestrogen patches (i.e. Estraderm, Estradot)

🞏 Oestrogen gels (i.e Sandrena)

🞏 Oestrogen implants

🞏 Oestrogen/progesterone oral combined contraceptive pill (i.e.Microgynon, Levlen, Estelle)

🞏 Spironolactone (i.e. Spiractin, Aldactone)

🞏 Cyproterone acetate (i.e. Androcur, Anterone, Cyprocur, Cyprone, Cyporstat, Cyproterone)

🞏 Bicalutamide (Cosudex)

🞏 GnRH analogues ("puberty blockers", Eligard, Zoladex)

🞏 Progestins (i.e. Provera, Ralovera)
🞏 Other (please specify) ____________

1. Have you ever taken any hormonal treatments without a prescription (for example from the internet or from a friend's supply)?

🞏 Yes
🞏 No
🞏 Prefer not to say

1. Have you experienced any difficulty accessing hormonal treatment? (select all that apply)

🞏 None
🞏 Unable to find a doctor to prescribe

🞏 Unable to afford costs of prescriptions

🞏 Unable to afford cost of doctors’ appointments

🞏 Pathway to accessing hormones was too difficult

🞏 Other (please specify) __________________

1. Do you feel that a mental health assessment for trans and gender diverse individuals should be performed prior to accessing hormonal treatment?

🞏 Yes, in all cases
🞏 Sometimes or in some circumstances

🞏 No
🞏 Unsure/prefer not to say

1. Gender-affirming surgery can be challenging to access in Australia. Have you had, or do you desire any of the following procedures?

|  | Have had | Want someday | Don’t want |
| --- | --- | --- | --- |
| Breast mastectomy ("top surgery") |  |  |  |
| Breast augmentation |  |  |  |
| Genital surgery ("bottom surgery") |  |  |  |
| Genital surgery ("bottom surgery") |  |  |  |
| Voice surgery |  |  |  |
| Other (please specify) |  |  |  |

1. Have you ever had any gender-affirming surgery performed overseas? (in a country outside of Australia)

🞏 Yes
🞏 No
🞏 Unsure/prefer not to say

1. Have you ever been medically diagnosed with any of the following medical conditions? (select all that apply)

🞏 Depression
🞏 Anxiety
🞏 Autism spectrum or Asperger's syndrome
🞏 ADHD
🞏 Bipolar disorder
🞏 Ischaemic heart disease
🞏 Stroke
🞏 Emphysema
🞏 Blood clots (pulmonary embolus or deep vein thrombosis)

🞏 Cancer
🞏 HIV/AIDS
🞏 Liver disease
🞏 Diabetes mellitus
🞏 Fractures (broken bone)
🞏 Kidney or renal disease

1. Have you ever intentionally self-harmed?

🞏 Yes

🞏 No
🞏 Prefer not to say

1. Have you ever attempted suicide?

🞏 Yes

🞏 No
🞏 Prefer not to say

**Section 3: Barriers to healthcare**

1. In the past 12 months how any times have you visited a doctor (for your own health)?

🞏 0
🞏 1-2 times

🞏 3-5 times

🞏 6-10 times
🞏 11-20 times
🞏 More than 20 times

1. Do you feel confident about asking your family doctor/GP questions and discussing health issues that concern you?

🞏 Totally confident

🞏 Somewhat confident

🞏 Not very confident

🞏 Not confident at all

1. Are you a member of any trans support groups (including social media based)?

🞏 Yes

🞏 No

🞏 Unsure/prefer not to say
What groups are you a member of? (optional) _____________________

1. Do you have any particular websites that you use to access information about trans health (e.g. quora, wikipedia)?

🞏 Yes
🞏 No
🞏 Unsure/Prefer not to say

What websites do you use? (optional) _____________________

1. Do you use social media (e.g. twitter, Facebook)?
   🞏 Yes, daily

🞏 Yes, a couple of times a week

🞏 Yes, occasionally (e.g. once a week)

🞏 Yes but rarely (e.g. once a fortnight)

🞏 Not at all

1. Please rank the options below indicating the way you prefer to receive information about trans health (where 1 is your most preferred, and 9 is your least preferred).

🞏 Online (websites, emails etc)
🞏 Videos or podcasts
🞏 Hardcopy print materials (e.g. brochures, booklets)

🞏 Small local community talk/ seminars
🞏 Larger group gathering (e.g. conferences, exhibitions)

🞏 Online group forums (e.g. webinars)
🞏 Social media (e.g. facebook)
🞏 Telephone contact
🞏 Apps (on mobile devices)

1. Because of your trans status have you ever experienced any of the following (select all that apply)?

🞏 Discrimination from employment (i.e. lost a job or overlooked for a job)

🞏 Discrimination from housing (i.e. denied a rental application)

🞏 Discrimination from accessing healthcare
🞏 Discrimination from government services (i.e. Centrelink)

🞏 Physical assault

🞏 Verbal abuse

🞏 Domestic violence

🞏 None

Please feel free to comment (optional)

1. Would you find an online website with Australian-based reliable trans and gender diverse health resources beneficial? This would include information on medical transition, legal transition, gender health services in Australia etc.

🞏 Yes
🞏 No

🞏 Unsure

What health-related content would you find most useful? ___________________

1. If you had to choose one, where do you think government funding in transgender health should be directed?

🞏 Education about gender diversity (i.e. raising awareness in the community/schools/workplaces)

🞏 Gender clinics
🞏 Support groups
🞏 Trans advocacy groups

🞏 Better training for doctors in trans issues

🞏 Trans/gender medical research

🞏 Psychology/psychiatry services

🞏 Counselling

🞏 Other (please specify)

1. How do you think healthcare professionals can better support you?
2. What do you think are the two most important issues for your health i.e. If you could improve your health right now, what would you do?
3. Are there any areas of trans-related medical research that you would like addressed?
4. Do you have any other comments?

**Has this survey raised concerns for you or someone you know?**

**Help is available by making an appointment with your doctor or by contacting the following organisations:
Lifeline on 13 11 14 or online at www.lifeline.org.au.
Beyond Blue on 1300 22 4636 or https://www.beyondblue.org.au**

**Q Life, (LGBTI-specific support service)1800 184 527 or qlife.org.au**

**We are keen to hear from as many trans and gender diverse individuals in Australia as possible. Please feel free to share this survey link with your trans and gender diverse friends and groups in Australia.**
